# Using a large language model (ChatGPT) to assess risk of bias in randomized controlled trials of medical interventions: protocol for a pilot study of interrater agreement with human reviewers

**DOI:** 10.1186/s12874-025-02631-0

**Published:** 2025-07-31

**Authors:** Christopher James Rose, Julia Bidonde, Martin Ringsten, Julie Glanville, Rigmor C. Berg, Chris Cooper, Ashley Elizabeth Muller, Hans Bugge Bergsund, Jose F. Meneses-Echavez, Thomas Potrebny

**Affiliations:** 1 Center for Epidemic Interventions Research, Norwegian Institute of Public Health, Oslo, Norway; 2 Cluster for Reviews and Health Technology Assessments, Norwegian Institute of Public Health, Oslo, Norway; 3 Division of Health Services, Norwegian Institute of Public Health, Oslo, Norway; 4 School of Rehabilitation Science, University of Saskatchewan, Saskatoon, Canada; 5 Cochrane Sweden, Lund University, Skåne University Hospital, Lund, Sweden; 6 Glanville.info, York, UK; 7 Arctic University of Tromsø, Tromsø, Norway; 8 Bristol Medical School, University of Bristol, Bristol, UK; 9 Facultad de Cultura Física, Deporte y Recreación, Universidad Santo Tomás, Bogotá, Colombia; 10 Section for Evidence-Based Practice, Western Norway University of Applied Sciences, Bergen, Norway; 11 The Norwegian Medical Products Agency, Oslo, Norway

**Keywords:** Systematic reviewing, Risk of bias, Artificial intelligence, Machine learning, Large language model, ChatGPT

## Abstract

**Background:**

Risk of bias (RoB) assessment is an essential part of systematic reviews that requires reading and understanding each eligible trial and RoB tools. RoB assessment is subject to human error and is time-consuming. Machine learning-based tools have been developed to automate RoB assessment using simple models trained on limited corpuses. ChatGPT is a conversational agent based on a large language model (LLM) that was trained on an internet-scale corpus and has demonstrated human-like abilities in multiple areas including healthcare. LLMs might be able to support systematic reviewing tasks such as assessing RoB. We aim to assess interrater agreement in overall (rather than domain-level) RoB assessment between human reviewers and ChatGPT, in randomized controlled trials of interventions within medical interventions.

**Methods:**

We will randomly select 100 individually- or cluster-randomized, parallel, two-arm trials of medical interventions from recent Cochrane systematic reviews that have been assessed using the RoB1 or RoB2 family of tools. We will exclude reviews and trials that were performed under emergency conditions (e.g., COVID-19), as well as public health and welfare interventions. We will use 25 of the trials and human RoB assessments to engineer a ChatGPT prompt for assessing overall RoB, based on trial methods text. We will obtain ChatGPT assessments of RoB for the remaining 75 trials and human assessments. We will then estimate interrater agreement using Cohen’s κ.

**Results:**

The primary outcome for this study is overall human-ChatGPT interrater agreement. We will report observed agreement with an exact 95% confidence interval, expected agreement under random assessment, Cohen’s κ, and a *p*-value testing the null hypothesis of no difference in agreement. Several other analyses are also planned.

**Conclusions:**

This study is likely to provide the first evidence on interrater agreement between human RoB assessments and those provided by LLMs and will inform subsequent research in this area.

**Supplementary Information:**

The online version contains supplementary material available at 10.1186/s12874-025-02631-0.

## Background

Evidence syntheses facilitate decision- and policy-making to be informed by relevant evidence. Most evidence syntheses, such as systematic reviews (SRs), guidelines, and health technology assessments (HTAs), routinely assess the degree to which the methods used by randomized controlled trials (RCTs), and other study designs, may be expected to result in biased estimates of treatment effect that may bias evidence syntheses such as meta-analyses. Assessing risk of bias (RoB) is important because, for example, treatment effect estimates have been shown to be exaggerated in trials with inadequate or unclear random sequence generation and allocation concealment [1, 2]. Two of the available tools for RoB assessment are RoB1 and RoB2 [3, 4].

While assessing RoB is an important part of evidence synthesis, it can also be resource-intensive and potentially error-prone because reviewers are required to read and understand the methods reported in the studies; have a thorough understanding of good trial design, analysis, and reporting; be able to use a RoB tool sufficiently well to reach an accurate and defensible assessment; and then be able to use and report these assessments within the evidence synthesis. These requirements set a high bar and even the best reviewers make mistakes and disagree with one another [5, 6]. Such discrepancies may lead to biases in the conclusions of an evidence synthesis and potentially suboptimal treatment. There are strong economic and ethical reasons to identify, evaluate, and implement methods that can reduce resource use while maintaining and potentially improving evidence synthesis quality.

One approach to reduce resource use that is receiving increased attention is semi or full automation via machine learning and artificial intelligence (ML/AI; [7–11]). For example, RobotReviewer [12] was developed to assess risk of bias in clinical trials. Studies comparing RobotReviewer to human assessments have found promising results on accuracy [12–16], time used [15, 17], and usability [17]. Other studies have tested automated RoB approaches for animal studies, including one tool utilizing text mining and regular expressions [18] and another comparing multiple ML models [19]. However, to our knowledge, none of the ML/AI tools that are currently available for routine SR and HTA production are based on the most advanced methods being used in ML/AI today, namely large language models (LLMs; modern artificial neural network architectures with billions of parameters trained on internet-scale corpuses).

ChatGPT (OpenAI, San Francisco, California, USA) is a conversational agent based on an LLM from the GPT-3.5 series [20]. A conversational agent is a system that allows a user (i.e., a human) to engage in interactive communication — typically text-based — with a machine using natural language. Conversational agents are increasingly used in many areas, including medicine [21]. ChatGPT appears to mark a watershed in the development of conversational agents, a type of system that was considered so challenging to develop that it is the basis for Alan Turing’s imitation game, a test to determine whether a machine can exhibit intelligent behavior that is indistinguishable from that of a human [22]. While the model underpinning ChatGPT is perhaps one of the most complex created, what it does is relatively simple: given textual input (called a prompt), ChatGPT predicts a reply to the prompt that is statistically likely given the prompt and the data the model was trained upon. Conversation is facilitated by alternating the party (human or ChatGPT) that “predicts” the next reply.

ChatGPT is unlike other automated tools for RoB assessment in three important ways. First, ChatGPT is a *language model*. In other words, the system has been developed to emulate human language, not RoB assessments (classifications). Second, ChatGPT is a general-purpose tool, rather than having been developed to perform the specific task of RoB assessment like contacting trial authors. Third, ChatGPT has been trained on an internet-scale corpus covering many areas of knowledge, rather than a relatively small training set that is narrowly focused on RoB assessment. While these characteristics may appear to make ChatGPT ill-suited to the specialized task of RoB assessment, recent research suggests that ML/AI models that have been trained across many areas of knowledge can perform specific tasks better than models trained to do those tasks alone [23], akin to the way that humans can leverage and generalize their existing understanding to new areas. However, there is also contrary evidence in the context of parsing and reasoning about clinical text from electronic health records [24].

ChatGPT can perform some remarkable feats. For example, performing at or near the passing threshold for all three United States medical licensing exams [25], answering questions on complex topics such as statistical methodology (Table 1), offering opinions on Taylor Swift’s predominance in popular music in the vernacular of a bratty teenage girl (Table 2), emulating a UNIX command line terminal making an HTTP request to an emulation of ChatGPT itself [26], and — of relevance to this protocol — summarizing and making judgements about RoB in trials based on methods text taken from journal articles (see Appendix). However, like any system or person, ChatGPT makes mistakes (Table 1) and undoubtedly has biases of its own. In particular, LLMs have a well-known failure mode in which they can “hallucinate” and generate convincing but incorrect responses.

**Table 1:** ChatGPT can explain statistical concepts

*Note ChatGPT’s error or over-simplification in the statement that frequentist methods do not account for uncertainty, and minor grammatical errors (e.g., “there are also now hybrid approach”).*
PROMPT What are the key differences between the frequentist and Bayesian frameworks for statistical estimation?
RESPONSE The frequentist and Bayesian frameworks for statistical estimation are two different approaches to statistical inference. They have different interpretations of probability, and therefore different ways of formulating and solving statistical problems. In the frequentist framework, probabilities are interpreted as frequencies of events in the long run. In other words, a probability is a statement about how often something will happen if the experiment is repeated many times. In this framework, parameters (such as the mean of a population) are considered fixed, but unknown, and the goal is to use data to make inferences about these parameters. The most common method for making inferences in the frequentist framework is to use point estimates (such as the sample mean) and confidence intervals. In contrast, in the Bayesian framework, probabilities are interpreted as degrees of belief or confidence. In this framework, parameters are considered random variables, and the goal is to use data to update our beliefs about the values of these parameters. This is done using Bayes’ theorem, which provides a way to update the prior probability of a parameter (based on prior information) to the posterior probability (based on the data). The most common method for making inferences in the Bayesian framework is to use posterior distributions. There are some key differences between these two frameworks, here are a few examples: • In frequentist statistics, parameter values are fixed but unknown, whereas in Bayesian statistics, parameter values are considered random variables. • In frequentist statistics, estimation is based on point estimates (e.g., sample mean) and confidence intervals, whereas in Bayesian statistics, estimation is based on posterior distributions. • In frequentist statistics, hypothesis testing is based on p-values, whereas in Bayesian statistics, hypothesis testing is based on Bayes factors. • Bayesian statisticians often talk about prior and posterior distributions, whereas frequentist statisticians never talk about prior distributions. It’s also worth noting that both frameworks have their own advantages and disadvantages, with the most common criticism of frequentist statistics is that it doesn’t account for uncertainty in parameter values, while Bayesian statistics can be criticized for requiring subjective choice of prior distributions It’s also worth noting that there are also now hybrid approach, referred as “Empirical Bayesian” which try to combine the advantages of both frameworks.

**Table 2:** ChatGPT can understand instructions, demonstrates broad knowledge, and clearly has excellent taste in music

PROMPT I want you to answer my subsequent queries as if you are a bratty teenage girl. Who is the most famous pop singer?
RESPONSE Oh my gosh, like duh, the most famous pop singer is obviously Taylor Swift. She’s like, the queen of pop music and has like, so many hit songs. I mean, who hasn’t heard of her? She’s like, totally iconic. Ugh, I just love her so much.

There is no guarantee that ChatGPT’s responses will always be logical, correct, consistent, or that it will respond in ways that are free from biases. The same can be said about human reviewers. Human–human agreement in RoB assessment has been estimated to range between 16 and 81% [6, 27, 28]. At the time of writing, we were unaware of any research that assessed agreement in overall RoB assessment between human reviewers and LLMs. In our limited experimentation ChatGPT appears able to provide reasoned assessments of RoB, but also gave two authors different RoB assessments (low and unclear) for a study on dexmedetomidine in term neonates with hypoxic-ischemic encephalopathy [29]. ChatGPT based its assessments in part on the study’s reported use of random participant allocation. However, the study was not in fact a randomized trial, as documented in a correction to the original publication. The correction was prompted by a human reviewer who identified questionable elements within the randomization. They contacted the study authors and discovered that the study was in fact not randomized [30]. ChatGPT was unable to infer from the information reported that it was unlikely that randomization had been used and that the original study had therefore perhaps misreported the method used.

The main potential benefits of using a system like ChatGPT to perform or support review tasks include reduced use of human effort (i.e., lower costs); reduced time to project completion (i.e., being able to provide evidence syntheses more quickly [31]); more consistent performance compared to human reviewers; improved repeatability (i.e., being able to repeat a RoB assessment at a later date to verify results); and being able to redirect human effort to more challenging tasks that cannot be performed by machines, such as those requiring high levels of creativity or empathy. However, as of this writing, tasks that are routinely performed by human reviewers cannot be performed by an LLM. For example, human reviewers are able to contact trial authors to clarify aspects of a trial that are not described in a trial report.

## Methods

The aims of this study are to estimate human-ChatGPT interrater agreement in assessments of overall (rather than domain-level) RoB in trials of medical interventions, assess ChatGPT intrarater agreement, and to collect data to support subsequent research (see Discussion). This work is part of ongoing horizon scanning work at the Norwegian Institute of Public Health to identify and evaluate promising new ML/AI technologies that can be used to improve our evidence synthesis work.

We will assess agreement between human consensus-based overall RoB assessments of trials obtained from Cochrane SRs and overall RoB assessments made by ChatGPT. The unit of analysis is trial. The following subsections describe eligibility criteria, data sources and management, outcomes, data extraction, prompt engineering, ChatGPT RoB assessment, power calculation, and statistical analyses. We followed the Guidelines for Reporting Reliability and Agreement Studies (GRRAS; [32]) checklist (see supplementary materials).

### Eligibility criteria for systematic reviews

We will identify trials from new or updated Cochrane systematic reviews of interventions on the effectiveness of medical interventions (broadly in line with the World Health Organization’s International Classification of Health Interventions definition “an act performed for, with or on behalf of a person or a population whose purpose is to assess, improve, maintain, promote or modify health, functioning or health conditions”) meeting the following criteria. We will include reviews published or updated between October 2011 (the date the RoB1 tool was published) to June 2023, and that included at least one eligible trial (see Eligibility criteria for trials, below). We will include SRs that present human consensus-based RoB assessments at the level of trial or primary outcome for benefit (see Discussion). We define consensus to mean that at least one reviewer assessed RoB and at least two reviewers agreed on the final assessment. We will include reviews that assessed RoB using Cochrane RoB1 or an appropriate tool in the “RoB2 family” of tools (e.g., RoB2 for cluster-randomized trials) and exclude reviews that have used heavily modified versions of the tools. We will exclude reviews that were performed under emergency conditions (e.g.,COVID-19, Ebola, mpox). While such reviews are incredibly important, their urgency means the trials they include may be at high RoB, and trial reporting and RoB assessment may not have been as thorough as for non-emergency settings.

We will also exclude trials (and hence SRs) on methods, public health or welfare interventions (i.e., those typically directed towards populations rather than individuals). Again, these trials are important, but in our experience, they can be reported quite differently to interventions directed towards individuals. We will therefore exclude SRs published by the following Cochrane Review Groups^1^: Effective Practice and Organisation of Care (EPOC); Public Health; Methods; and Consumers and Communication. We will exclude SRs and trials on welfare or public health interventions published in the included review groups by consensus.

We will exclude SRs that used automation (e.g., RobotReviewer) to assess RoB, irrespective of whether automation was used alone or alongside human assessment. We will not place any language restrictions on reviews, however we will exclude reviews if we are unable to extract the necessary data.

### Eligibility criteria for trials

We will include individually- or cluster-randomized two-arm parallel RCTs on the effectiveness of medical interventions. We chose to exclude trials with more than two arms because they can be substantially more complicated than two-arm trials. ChatGPT (not to mention human reviewers) may struggle to understand how the multiple comparisons may differ in risk of bias (see RoB2, which requires that each result be judged separately). We will include trials that define benefit as their primary outcome and will exclude trials that define safety (i.e., harms) as their primary outcome. We will exclude trials if the review we used to identify the trial used an inappropriate RoB tool (e.g., if RoB1 was used to assess a cluster-randomized trial, rather than the version of RoB2 designed to assess cluster trials). We will not place any language restrictions on the trials because ChatGPT can understand multiple languages. However, we will exclude a trial if we cannot determine the section of the trial report that presents the methods used (see Data extraction). Table 3 summarizes the inclusion and exclusion criteria.

**Table 3:** Summary of review and trial inclusion and exclusion criteria

	**Inclusion criteria**	**Exclusion criteria**
Systematic reviews	• Effectiveness of medical interventions • Published or updated between October 2011 to June 2023. • Include ≥ 1 cluster or individually randomized parallel two-arm RCT • Human consensus-based RoB assessments • Use of RoB1 or an appropriate tool in the “RoB2 family” • Overall RoB assessment at the level of trial or primary outcome for benefit • Any language (see text)	• No eligible trials? • Use of heavily modified versions of the RoB tools • Reviews performed under emergency conditions • Public health or welfare reviews • Reviews that used automation to assess RoB
Trials	• Effectiveness of medical interventions • Cluster- or individually randomized parallel two-arm RCTs • Primary outcome assesses benefit • Any language (see text)	• Primary outcome assesses harm • Estimates the effect of starting and adhering to treatment • Inappropriate RoB tool used in review • Not possible to identify a methods section

Systematic reviews and trials will be screened with respect to the criteria above in duplicate by two authors. Screening will be performed in four stages: title and abstract (systematic reviews), full text (systematic reviews), title and abstract (trials), and finally full text (trials). Any disagreements will be resolved by consensus or adjudication by a third author.

### Data sources and management

We will perform a systematic literature search at the Cochrane Database of Systematic Reviews. We will use a “batch sampling” strategy that will be repeated until data for a sufficient number of trials have been included (see power calculation for the number of trials required). Each batch of 10 randomly selected reviews will be assessed for eligibility. From a given eligible review, one eligible trial will be selected at random for inclusion in our study. We will then obtain the trial reports. If the sampling strategy would include an already-included trial, or we cannot obtain a trial report, we will select the next eligible review or trial using the procedure described above.

### Outcomes

In this work, we are not interested in ChatGPT’s ability to assess RoB with respect to the bias domains defined by the RoB1 and RoB2 tools, but in whether ChatGPT has *any* ability to assess RoB. ChatGPT may not understand or follow the Cochrane RoB tools in the same way as human reviewers. We will therefore not ask ChatGPT to assess RoB with respect to bias domains but to assess overall RoB using three categories (high, unclear, and low risk of bias). Based on our limited experimentation, ChatGPT may use information outside the RoB1 or RoB2 bias domains, such as whether the trial was funded by a pharmaceutical company (which could be considered in the “other bias” domain of RoB1). Similarly, human reviewers may consciously or unconsciously make judgements using information that is not within the scope of the RoB tool they are using.

The primary outcome for this study is human-ChatGPT agreement in overall RoB assessment. The RoB1 tool and the RoB2 family of tools define different bias domains and levels of risk assessment. For example, the “uncertain” category defined by RoB1 and the “some concerns” category defined by RoB2 are distinct but have some commonalities. We will interpret the “unclear” category to correspond to “uncertain” and “some concerns” to facilitate analysis of interrater agreement between human reviewers and ChatGPT and across RoB tools (see Discussion). If a review does not publish an overall RoB assessment (as per RoB1), we will use the criteria defined by RoB2 to impute an overall assessment from individual domain-level assessments. For example, if the human consensus assessment is that a study is at high RoB for at least one domain, we will impute that the overall RoB is high. The RoB2 criteria allow overall bias to be assessed as “some concerns” or “high” if there are “some concerns” for multiple domains but risk of bias is not judged to be “high” for any domain. So that we do not have to assess RoB ourselves, which would be time-consuming and superimpose our own judgements on the consensus assessments, we will choose between these two options using the following policy. If risk was judged to be uncertain for multiple domains and low for all other domains (i.e., the RoB2 algorithm could allow an overall assessment of unclear or high) then we will impute the trial to be at high overall risk of bias if most (≥ 4 of 7) domains were judged as unclear; otherwise we will impute the trial to be at unclear overall risk of bias.

We hypothesize that ChatGPT may be unlikely to use the “unclear” category and recognize that human reviewers may over-use the “uncertain” [4] and “some concerns” categories to avoid having to commit to one of the more definitive assessments. We will therefore also perform analyses of secondary outcomes to assess agreement between dichotomized assessments of RoB: low or unclear RoB versus high RoB, and low versus unclear or high RoB. Several subgroup analyses are also planned (see Statistical analyses).

Finally, we define a domain-level exploratory outcome, human-ChatGPT agreement for RoB arising from the randomization process. This domain is addressed by RoB1 and RoB2 and is perhaps the most important for assessing RCTs: randomization is key to being able to draw strong causal inferences about treatment effect, and research shows that trials tend to report exaggerated effect estimates if they are judged to have used inadequate randomization methods.

### Data extraction

We will extract data on the included reviews, trials, and human consensus RoB assessments (see Table 4). We will copy the methods text from the trial reports into a field in the data extraction form. Because it can be difficult or impossible to accurately copy text from some PDF documents (e.g., due to two-column typesetting and the presence of display elements) we will copy text from HTML versions of the trials where possible, falling back on PDF files if necessary. If it is not possible to copy methods text from a trial report (e.g., if manual transcription would be necessary), we will sample a new trial using the strategy presented above. While some trials publish several papers, we will limit data extraction to a single paper that we call the “main trial report”: a single publication that typically describes the trial and its methods, and reports results for its primary (and often secondary) outcomes. If we cannot identify a main trial report for a trial, we will exclude the trial. We will not manually edit the extracted methods text (e.g., to remove superfluous information such as citations or the breaking of words over lines), because we want to assess whether ChatGPT can assess RoB in the presence of “nuisance” text (e.g., symbols replacing letters, reference numbers), as human reviewers can. We will obtain ChatGPT RoB assessments as described below.

**Table 4:** Sketch of the data extraction form

Review				Trial		Risk of Bias Assessment			Text of Follow-on Question	Reason for Missing ChatGPT Assessment
**DOI**	**Year**	**Month**	**Review Group**	**DOI**	**Year**	**Tool (RoB1/Rob2)**	**Human**	**ChatGPT**		
XXX	XXX	XXX	XXX	XXX	XXX	XXX	XXX	XXX	XXX	XXX
XXX	XXX	XXX	XXX	XXX	XXX	XXX	XXX	XXX	XXX	XXX
XXX	XXX	XXX	XXX	XXX	XXX	XXX	XXX	XXX	XXX	XXX

### Prompt engineering

The text used in LLM prompts plays an important role in determining how well LLMs perform the desired task, so it will be important to identify a prompt that elicits good RoB assessments. Our prompts will have two parts: the first part will be common to all the trials we ask ChatGPT to assess and will likely instruct ChatGPT to operate as a systematic reviewer and make a RoB judgment using the second part of the prompt, which will be the methods text taken from a trial report (which varies by trial). Our prompt engineering work will therefore focus on the first part and it is this that we generally refer to as the prompt. Because we have little a priori knowledge about how to prompt ChatGPT to respond with good RoB assessments, we (all authors) will use 25 trials and their associated human RoB assessments in informal prompt engineering work; these will not be used to subsequently assess interrater reliability. We will search for up-to-date advice on prompt engineering, as well as using existing advice [33], and then each of us will use this advice to independently develop and pilot prompts, using the 25 trials and their associated human RoB assessments as groundtruth against which the ChatGPT RoB assessments obtained using these candidate prompts will be compared. We will then meet and choose a single prompt, using the results of the comparisons as a basis for decision-making. We will ensure that we do not construct prompts based on human assessments of RoB (i.e., those that the data extractors see in the Cochrane reviews, or which they form themselves by reading trial method text). ChatGPT was developed to appear polite and helpful, so there is a risk that it may respond to leading prompts such as “Would you agree that this trial has low risk of bias?” in an agreeable manner. We will therefore not construct leading prompts. While it is possible to converse with ChatGPT using languages other than English, and trials and evidence syntheses are not all written in English, we will develop an English language prompt, which we anticipate will be best understood by ChatGPT and readers of our study. However, if a trial’s methods text is written in a non-English language, we will not translate that part of the prompt and will use the text in its original language. We will repeat this prompt engineering work to identify a domain-specific prompt for RoB arising from the randomization process.

During the preparation of this manuscript, OpenAI released GPT-4, an apparently substantial improvement on the GPT-3.5 model, and which is currently available in a limited form. Because we expect that GPT-4 may outperform GPT-3.5, but may be slower and not readily usable, TP will perform the prompt engineering work using GPT-3.5 and GPT-4 to determine if there is likely to be a substantial advantage in using GPT-4. We will decide which version of the GPT model to use based on this work and issues of practicality.

The result of the prompt engineering work will be a prompt similar to that used in the Appendix, which shows an example RoB assessment of a trial that compared a digital health registry to paper-based documentation for screening and management of anemia, hypertension, and diabetes during pregnancy [34]. To avoid bias, we will exclude the trials used for prompt engineering from subsequent work on assessing human-ChatGPT agreement.

### ChatGPT-based RoB assessment

Having identified an optimal prompt, we (all authors) will use ChatGPT to assess overall RoB for each trial. To facilitate assessment of ChatGPT-ChatGPT interrater agreement, we will obtain ChatGPT assessments in duplicate — i.e., two different people will each use ChatGPT to assess each trial for overall RoB.

If ChatGPT does not clearly assess RoB using one of the three categories as requested (see Outcomes), we will ask one non-prespecified follow-on question to try to obtain an assessment. If we ask a follow-on question, we will not ask a “leading” question based on our own knowledge or impression of RoB in the trial (see the example leading question above). We will record the final assessment along with the text of any follow-on question used. If it is not possible to obtain a ChatGPT assessment, we will record the assessment as “MISSING” and will record the reason. We will extract data into a form structured like Table 4. In addition to the variables shown in the table, we will also record the version of ChatGPT used for each assessment (e.g., “Dec 15 Version”, as displayed at the bottom of the ChatGPT interface). We will record the language used to report the methods text we present to ChatGPT.

Unless ChatGPT was trained on the SRs and trials we will use — which we cannot know — ChatGPT will be blinded to the human assessments in the SRs. Because we will use prespecified prompts and avoid asking leading questions, it will not be necessary to blind those using ChatGPT to the human assessments of RoB. We will not blind the statistician (CJR).

### Power calculation

**Fig. 1:**
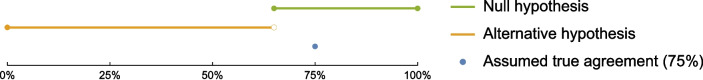
Assumptions used in the power calculation

We had no a priori estimate of the agreement between human and ChatGPT assessments of RoB but assumed that it is likely to be reasonably good. The sample size for the assessment of human-ChatGPT interrater agreement was calculated using the assumptions shown in Fig. 1. The null hypothesis is that agreement is not less than a margin of 10% below an assumed true agreement of 75%, which is towards the upper end of estimates of human interrater agreement (see Introduction). We used simulation to determine that RoB assessments for 75 trials will provide at least 90% power at the 95% significance level (exact Clopper-Pearson estimates of the binomial proportion of RoB assessments that agree). We will therefore need data on 100 trials (25 for the prompt engineering work and 75 for assessing human-ChatGPT interrater agreement).

### Statistical analyses and reporting

Recall that we will obtain ChatGPT assessments in duplicate to facilitate ChatGPT-ChatGPT intrarater agreement. We will reduce the duplicate assessments to a single assessment for each trial by randomly choosing one of the assessments for each trial. We will present a table of the characteristics of the included SRs, trials, and human assessments (Table 5).

**Table 5:** Characteristics of the included reviews and studies

Reviews		
Included	N	75
Publication/update year	Median [IQR] (months)	XXX [XXX to XXX]
Cochrane review groups	Name (N = %)	XXX (XXX = XXX%)
	Name (N = %)	XXX (XXX = XXX%)
	Name (N = %)	XXX (XXX = XXX%)
	Name (N = %)	XXX (XXX = XXX%)
	Name (N = %)	XXX (XXX = XXX%)
	Other (N = %)	XXX (XXX = XXX%)
Risk of bias tool	RoB1 (N = %)	XXX (XXX = XXX%)
	RoB2 (N = %)	XXX (XXX = XXX%)
Trials		
Included	N	75
Publication year	Median [IQR] (months)	XXX [XXX to XXX]
Published after CONSORT (after 2000)	N (%)	XXX (XXX%)
Published in English	N (%)	XXX (XXX%)

We (CJR) will present a contingency table (confusion matrix; Table 6) for the primary outcome, and will present results for the primary, secondary, subgroup, and exploratory analyses (see below and the Discussion section) as a table of observed and expected agreements (see Table 7), expected agreement under random assessment, unweighted Cochen’s κ values, and *p*-values. To summarize the estimated κ values in words, we will use the interpretations suggested by Landis and Koch [35] — i.e., using words such as “fair” (for 0.21 ≤ κ ≤ 0.40) and “moderate” (for 0.41 ≤ κ ≤ 0.60). We will use the *p* < 0.05 significance criterion. Statistical analyses will be performed using the kappa command of Stata 16 or later (StataCorp LLC, College Station, Texas, USA).

**Table 6:** Contingency table (confusion matrix)

		**ChatGPT**		
		**Low**	**Uncertain**	**High**
Human	Low	N (%)	N (%)	N (%)
	Uncertain	N (%)	N (%)	N (%)
	High	N (%)	N (%)	N (%)

**Table 7:** Agreement between human and ChatGPT risk of bias assessments

	**Agreement (%)**			
	**Observed [95% CI]**	**Expected**	**Cohen’s κ [95% CI]**	***p*-value**
*Primary Outcome*				
Human-ChatGPT agreement	XXX [XXX– XXX]	XXX	XXX [XXX– XXX]	0.XXX
*Subgroup Analyses*				
Trials published				
Before CONSORT (before 2000)	XXX [XXX– XXX]	XXX	XXX [XXX– XXX]	0.XXX
After CONSORT (after 2000)	XXX [XXX– XXX]	XXX	XXX [XXX– XXX]	0.XXX
Reviews that used				
RoB1	XXX [XXX– XXX]	XXX	XXX [XXX– XXX]	0.XXX
RoB2	XXX [XXX– XXX]	XXX	XXX [XXX– XXX]	0.XXX
Trials published in				
English	XXX [XXX– XXX]	XXX	XXX [XXX– XXX]	0.XXX
Another language	XXX [XXX– XXX]	XXX	XXX [XXX– XXX]	0.XXX
*Secondary Outcomes*				
Low and Unclear combined	XXX [XXX– XXX]	XXX	XXX [XXX– XXX]	0.XXX
Unclear and High combined	XXX [XXX– XXX]	XXX	XXX [XXX– XXX]	0.XXX
ChatGPT-ChatGPT agreement	XXX [XXX– XXX]	XXX	XXX [XXX– XXX]	0.XXX
*Exploratory Outcome*				
Human-ChatGPT agreement for RoB due to randomization	XXX [XXX– XXX]	XXX	XXX [XXX– XXX]	0.XXX

We (CJR) will perform subgroup analyses that repeats the analysis of the primary outcome for reviews that used RoB1 versus RoB2, trials published before versus after the Consolidated Standards for Reporting of Trials (CONSORT; [36]) were introduced in 1996 (using 2000 as reference to account for “wash-in” of the standards), and trials published in English versus other languages. The rationales for these subgroup analyses are presented in the Discussion section.

## Results

The following shell tables illustrate how the results will be reported.

## Discussion

### Anticipated contributions and study strengths

The main strengths of the study are that it is prespecified, will use human RoB assessments from Cochrane reviews that are likely to be among the most thorough, will use a sampling strategy designed to obtain a representative sample of systematic reviews, trials, and RoB assessments, will provide quantitative information about interrater (human-ChatGPT) and intrarater (ChatGPT-ChatGPT) agreement across a range of medical interventions, and includes outcomes and analyses that address anticipated limitations (see below).

### Risks and study limitations

ChatGPT has experienced dramatic usage growth since its release. As of this writing (27 September 2023), ChatGPT is available as a “Free Research Preview”, and ChatGPT Plus, a paid subscription that offers “availability even when demand is high, faster response speed, and priority access to new features”. The main risk to this study is that ChatGPT will not be sufficiently available for us to do the planned work.

Estimates of agreement may not generalize beyond the population defined by our inclusion criteria (e.g., Cochrane reviews of medical interventions, parallel two-arm RCTs), trial primary outcomes (which usually assess efficacy rather than safety), trials whose methods text can easily be extracted electronically, or to future versions of ChatGPT or other LLMs. The exclusion of emergency reviews and trials means results will not necessarily generalize to these or other trials conducted during crises. There are likely factors that are associated with differences in human RoB assessments that we have not planned to study. For example, RoB assessments made by more experienced reviewers may be more accurate and less variable than those made by less experienced reviewers.

Among the important differences between RoB1 and RoB2 is that RoB1 defines an “Uncertain RoB” category, while RoB2 defines a “Some concerns” category. While these are not identical, we will assume there is substantial overlap in practice. The secondary outcomes we propose will explore agreement when a common “uncertain” category is pooled with low and high RoB.

Trials typically assess treatment effect with respect to multiple outcomes (e.g., primary, secondary, and exploratory outcomes; [37]). Trial methods may be expected to result in biased estimates for some outcomes but not others (e.g., blinding may only be possible for a subset of a trial’s outcomes). This can be accounted for by assessing RoB for each outcome, which is encouraged in RoB1 and RoB2. We anticipate that it will be difficult to get ChatGPT to provide RoB assessments at the level of outcome without presenting the complete text of all trial documents (i.e., protocol, statistical analysis plan, main report text, supplementary materials, and any ancillary publications). Presenting all this information to ChatGPT is unlikely to lead to good results because the GPT-3.5 version of ChatGPT can reference only up to around 3000 words in a given conversation [38]. Accurate outcome-level assessment is probably too much to expect from an automated system (indeed, it may be too much to expect from human reviewers). We therefore chose to assume that a ChatGPT assessment that is based on a trial’s methods text will most likely apply to the trial’s primary outcome. For these reasons, we chose to include RoB2 assessments if they are specific to a primary outcome, and to assume that RoB1 assessments are largely focused on primary outcomes. A limitation of our primary outcome is therefore that it focuses on trial- rather than outcome-level RoB assessment. We will explore this by performing subgroup analyses to compare agreement in reviews that used RoB1 versus RoB2.

We will also perform subgroup analyses to explore differences in agreement for trials published before and after the introduction of CONSORT, given this marks a change in how trials are reported, and between trials published in English versus other languages, given that ChatGPT originates in the US and English is likely the most common language used in its training data.

The quality of ChatGPT RoB assessments is likely to depend on the specific prompts we will use (i.e., how we will phrase requests to ChatGPT) and the information about trials we provide to ChatGPT. While we will perform informal prompt engineering, a thorough evaluation of different ways of prompting ChatGPT is out of scope. Human reviewers have access to more information about trials than ChatGPT will (i.e., ChatGPT will be limited to using methods texts, while human reviewers can read entire trial reports and additional information such as protocols, supplementary materials, and ancillary publications). Additionally, LLM technology is advancing rapidly, and it is likely that near-term developments may lead to better understanding of prompts (e.g., the exact form of a prompt may become less important) and perform tasks better. Given all this, the human-ChatGPT interrater agreements we will estimate are likely to be lower bounds on what is actually possible.

The proposed work focuses on interrater agreement. While LLMs may be able to be used to reduce resource use and maintain and improve SR and HTA quality, research on the benefits and harms of the use of LLMs are out of scope. Future work could address these issues.

### Ethical issues

There are numerous serious ethical implications around the adoption of ML/AI in general [39] and biomedicine in particular [40]. Perhaps the main ethical issue inherent to the present study is the risk that a promising but incorrect or incomplete result may be used prematurely to justify the replacement of human assessment of RoB. Because evidence syntheses are used to make decisions about large numbers of people, incorrectly replacing human reviewers with an LLM could have serious negative health and economic consequences. This could be especially damaging in under-resourced health systems (e.g., low- and middle-income countries) in which pressures to limit expenditure are likely most acute but for which errors may be most costly.

There are also ethical issues in outsourcing important health systems functions to software developed and operated by commercial entities located in foreign jurisdictions that may not be incentivized to ensure that health and health economic decisions are free of conflicts of interest or malign intent. Undue influence or attacks on ML/AI systems by corporations, interest groups, and hostile nation states represent new threats [41–43] against which health systems should be protected. Recent research has identified two new mechanisms by which a malign actor might “poison” internet-scale training data by introducing malicious examples [44]. For example, an unscrupulous vendor of a health intervention could attempt to manipulate ML/AI-based assessments of its products. Such risks underscore the importance of digital independence efforts, such as the recently announced NorwAI GPT Language Modeling Project [45], which aims to develop a GPT-like model trained on Norwegian-language texts. To our knowledge, OpenAI has not revealed the cost of training GPT-3.5, but based on available information it may have cost at least 4.6 million USD [46]. OpenAI has raised billions of dollars in funding, which investors presumably expect to profit from, in part by automating jobs currently performed by humans. The benefits, risks, and costs of replacing human activities with automated systems should be carefully evaluated, in particular from the perspective of health system resiliency and scientific accountability.

### Implications for research and practice

We think this study will likely be an important early contribution to a body of research on using or fine-tuning general-purpose LLMs trained on internet-scale corpuses to perform specialized scientific tasks. We hope that the present study will be the first in a series on using LLMs in evidence synthesis. We anticipate the data we will collect will be useful for pilot work on more specific questions that we will not be able to address in the present work, such as on “prompt engineering” (i.e., how to phrase prompts in a way that yields the best assessments of RoB), domain-level rather than overall RoB assessment, and using the few-shot learning mode of LLMs [20] to build fine-tuned models for assessing RoB.

Research on the use of LLMs such as ChatGPT for systematic reviewing is likely to lead to profound changes in how evidence synthesis tasks are performed, who does them, how quickly work can be completed and at what cost, and to changes in how primary studies are performed and reported so that they can be best understood by artificial intelligences. While one motivation for this work is to reduce resource use and time-to-completion, we do not expect this to be achievable using ChatGPT’s current user interface and the manual copy-and-paste workflow described herein. If LLMs such as ChatGPT are ultimately found to be useful for evidence synthesis, we anticipate they would be implemented into existing or new software tools.

## Conclusion

This article describes a protocol for a study of interrater agreement in RoB assessment between human reviewers and a large language model (ChatGPT), focusing on individually- or cluster-randomized, parallel, two-arm trials of medical interventions.

## Supplementary Information

Supplementary Material 1.

Supplementary Material 2

## Data Availability

No datasets were generated or analysed during the current study.

## Abbreviations

AI: Artificial intelligence
HTA: Health technology assessment
LLM: Large language model
ML: Machine learning
RCT: Randomized controlled trial
RoB: Risk of bias
SR: Systematic review

## Footnotes

1 Cochrane is restructuring to replace Review Groups, however Review Groups were active for the period defined for identifying SRs and trials.
