# Supplementary material for: Using a large language model (ChatGPT) to assess risk of bias in randomized controlled trials of medical interventions: protocol for a pilot study of interrater agreement with human reviewers
Source: BMC Med Res Methodol. 2025 Jul 31;25:182. doi: 10.1186/s12874-025-02631-0 (PMC12315198; doi:10.1186/s12874-025-02631-0)
Supplement: Supplementary file 1 — Supplementary Material 1. [file 12874_2025_2631_MOESM1_ESM.docx]

## Appendix — Obtaining ChatGPT assessments of risk of bias

We will start a new ChatGPT “conversation” for each RoB assessment. This is necessary because ChatGPT conditions its responses on the history of the conversation. If we were to ask ChatGPT to assess one trial and then another (or the same trial) without starting a new conversation, it is possible ChatGPT will not correctly differentiate between the two distinct tasks. In the following, prompts to ChatGPT are shown in bold and italics, while ChatGPT’s responses are shown in italics. We will start the conversation with the prompt:

***In the following chat, I will provide text from the methods section of a RCT of an intervention. I want you to assess each text for risk of methodological bias using one of the following words: high, uncertain, low. At the end of the chat I want you to summarize risk of bias for the entire RCT.***

It is necessary to provide the text piecemeal because ChatGPT places a limit on the length of a prompt, so it is not possible to enter the text for an entire methods section in one prompt. After the prompt above, ChatGPT responded:

*I can certainly help you with that! Please go ahead and provide the text from the methods section of the RCT and I will assess the risk of methodological bias for each piece of text. At the end of the chat, I will provide a summary of the overall risk of bias for the entire RCT.*

We will then enter each paragraph of the methods section for the trial. For example, we may enter text such as the following, which was extracted from Venkateswaran 2022 ((34); for which CJR performed the statistical analysis) and is presented below as we would enter trial text into ChatGPT (e.g., without “cleaning” the text to remove citations). Note that it may be necessary to copy methods text from a field in the data extraction form and into a temporary file, in order to then copy and paste the distinct paragraphs into the ChatGPT user interface.

***This pragmatic, cluster-randomised, controlled, superiority trial was done in primary health-care clinics offering routine antenatal care in the West Bank, Palestine. The trial protocol with detailed methods and context has been published.17 In brief, the unit of randomisation was the public primary health-care clinic reporting to the Palestinian Ministry of Health. In the West Bank, there are about 400 public clinics providing routine antenatal care for low-risk pregnancies. Each clinic has a predefined referral clinic, and there are 86 referral clinics that provide care for pregnant women with certain risk factors or conditions. Private providers and the UN Relief and Works Agency for Palestine Refugees in the Near East (UNRWA) also offer antenatal care. About half of all pregnant women receive antenatal care in the public sector, and more than 80% deliver in government-run hospitals.***

In our testing, ChatGPT responded as follows:

*The risk of methodological bias in this study is low. The study is a cluster-randomized controlled trial, which is a strong design for minimizing bias. The study also clearly states the unit of randomization, which is the primary health-care clinic. Additionally, the study protocol has been published, which allows for transparency and allows other researchers to evaluate the methods used in the study.*

While ChatGPT did not follow our instruction exactly (i.e., it did not simply respond with “low”), it did provide an assessment for the text and also provided text that apparently explains the assessment. Few machine learning models are able to explain their decisions, which is legally required in some cases in some jurisdictions (e.g., see Recital 71 on the European Union’s General Data Protection Regulation). After entering all methods text for a trial, we will use the following prompt:

***What is your overall assessment of the risk of methodological bias? Please use one of the following words: high, uncertain, low.***

In the case of the Venkateswaran 2022 paper, ChatGPT responded:

*Overall, the risk of methodological bias in this RCT appears to be low. The study has taken appropriate steps to randomize the study participants, adjust for stratification and constraining variables, and minimize the risk of bias due to funding sources. Additionally, the study has a clear hypothesis, a clear plan for data analysis, and a sample size that is sufficient to detect the differences between the intervention and control groups. These are all important factors that can help to reduce the risk of methodological bias in a study.*

ChatGPT’s assessment for this study would therefore be recorded as “low”. Again, note that ChatGPT has provided an explanation, which includes details from the entire conversation (i.e., entire methods text).
